# Barriers to the acceptance of electronic medical records by physicians from systematic review to taxonomy and interventions

**DOI:** 10.1186/1472-6963-10-231

**Published:** 2010-08-06

**Authors:** Albert Boonstra, Manda Broekhuis

**Affiliations:** 1 Faculty of Economics and Business, University of Groningen, Groningen, The Netherlands

## Abstract

**Background:**

The main objective of this research is to identify, categorize, and analyze barriers perceived by physicians to the adoption of Electronic Medical Records (EMRs) in order to provide implementers with beneficial intervention options.

**Methods:**

A systematic literature review, based on research papers from 1998 to 2009, concerning barriers to the acceptance of EMRs by physicians was conducted. Four databases, "Science", "EBSCO", "PubMed" and "The Cochrane Library", were used in the literature search. Studies were included in the analysis if they reported on physicians' perceived barriers to implementing and using electronic medical records. Electronic medical records are defined as computerized medical information systems that collect, store and display patient information.

**Results:**

The study includes twenty-two articles that have considered barriers to EMR as perceived by physicians. Eight main categories of barriers, including a total of 31 sub-categories, were identified. These eight categories are: A) Financial, B) Technical, C) Time, D) Psychological, E) Social, F) Legal, G) Organizational, and H) Change Process. All these categories are interrelated with each other. In particular, Categories G (Organizational) and H (Change Process) seem to be mediating factors on other barriers. By adopting a change management perspective, we develop some barrier-related interventions that could overcome the identified barriers.

**Conclusions:**

Despite the positive effects of EMR usage in medical practices, the adoption rate of such systems is still low and meets resistance from physicians. This systematic review reveals that physicians may face a range of barriers when they approach EMR implementation. We conclude that the process of EMR implementation should be treated as a change project, and led by implementers or change managers, in medical practices. The quality of change management plays an important role in the success of EMR implementation. The barriers and suggested interventions highlighted in this study are intended to act as a reference for implementers of Electronic Medical Records. A careful diagnosis of the specific situation is required before relevant interventions can be determined.

## Background

Electronic Medical Records (EMRs) are computerized medical information systems that collect, store and display patient information. They are a means to create legible and organized recordings and to access clinical information about individual patients. Further, EMRs are intended to replace existing (often paper based) medical records which are already familiar to practitioners [1]. Patient records have been stored in paper form for centuries and, over this period of time, they have consumed increasing space and notably delayed access to efficient medical care [2]. In contrast, EMRs store individual patient clinical information electronically and enable instant availability of this information to all providers in the healthcare chain and so should assist in providing coherent and consistent care.

Electronic Medical Records (EMRs) and Electronic Health Records (EHRs) are viewed as interchangeable synonyms in most health informatics. Other similar expressions exist albeit with a sometimes slightly restricted focus. While EMRs have a general focus on medical care, Electronic Patient Records (EPRs) and Computerized Patient Records (CPRs) *"contain clinical information about a patients from a particular hospital" *and Electronic Health Care Records (EHCRs) *"contain a patient's health information" *[3].

The perceived advantages of EMRs can be summarized as *"optimizing the documentation of patient encounters, improving communication of information to physicians, improving access to patient medical information, reduction of errors, optimizing billing and improving reimbursement for services, forming a data repository for research and quality improvement, and reduction of paper" *[4]. As EMRs are viewed as having a great potential for improving quality, continuity, safety and efficiency in healthcare, they are being implemented across the world.

Despite the high expectations and interest in EMRs worldwide, their overall adoption rate is relatively low and they face several problems [5]. For instance, they are seen as contrary to a physician's traditional working style, they require a greater capability in dealing with computers and installing a system absorbs considerable financial resources [6]. According to Meinert [7], the slow rate of adoption suggests that resistance among physicians must be strong because physicians are the main frontline user-group of EMRs. Whether or not they support and use EMRs will have a great influence on other user-groups in a medical practice, such as nurses and administrative staff. As a result, physicians have a great impact on the overall adoption level of EMRs.

As it requires physicians to actively support and use EMRs to benefit from them, it is essential to understand the possible barriers to their implementation from the physicians' perspectives. Although there is already a body of literature on such barriers, there has been no systematic overview of these studies combined with an analysis of how to address these barriers. Therefore, the objective of this research is to identify, categorize, and analyze barriers perceived by physicians to the adoption of Electronic Medical Records (EMRs). Further, possible barrier-related interventions will be suggested to support implementers of EMRs in overcoming this reluctance.

In the following sections, a systematic literature review will be carried out to identify all the barriers that result in physicians showing resistance towards EMRs. Following this, these barriers will be categorized in a taxonomy in order to gain a wider understanding of them. From an analysis of the taxonomy, the relationships among these barriers will be highlighted. Finally, possible barrier-related interventions will be suggested that could reduce resistance, and further research opportunities identified.

## Methods

### Search Strategy

In order for this study to reflect recent events, be up-to-date and comprehensive, a systematic literature search of four relevant databases ("Web of Science", "EBSCO", "PubMed" and "The Cochrane Library") was conducted for the period from January 1998 to May 2009. The search was performed using the key words: "barrier", "physician", "doctor", |"electronic medical record", "electronic health record", "adopt*" and appropriate combinations thereof. Due to minor differences in search options, slightly different search strategies were used for each database. The reference lists of identified studies were scanned for further relevant articles.

On the database "Web of Science", the search was carried out according to the following search strategies:

*Search Strategy 1: *Key words (in the field of "Topic"): barrier* + Electronic Medical Record*

*Search Strategy 2: *Key words (in the field of "Topic"): barrier* + Electronic Health Record*

*Search Strategy 3: *Key words (in the field of "Topic"): physician* + Electronic Medical Record*

*Search Strategy 4: *Key words (in the field of "Topic"): physician* + Electronic Health Record*

For "Search Strategy 3" and "Search Strategy 4", the "Subject Areas" and "Document Types" fields were further refined in order to enhance the relevance of the results. The "Subject Areas" were limited to the sub-fields "Health Care Sciences & Services", "Medical Informatics", "Medicine", "General & Internal". "Document Types" were limited to "Article" and "Proceeding Paper".

On EBSCO, the search was based on the following two strategies:

*Search Strategy 5: *Key words (in the field of "TI Title"): barrier* + Electronic Medical Record*

*Search Strategy 6: *Key words (in the field of "TI Title"): barrier* + Electronic Health Record*

On "PubMed", the three search strategies for the initial collection of the literature were as follows:

*Search Strategy 7: *Key words (in the field of "Title"): barrier* + Electronic Medical Record*

*Search Strategy 8: *Key words (in the field of "Title"): barrier* + Electronic Health Record*

*Search Strategy 9: *Key words (in the field of "Title/Abstract"): adopt* + Electronic Health Record*

On "The Cochrane Library", the following strategies were used:

*Search Strategy 10: *Key words (in the field of "Title, Abstract or Keywords"): barrier* + Electronic Medical Record*

*Search Strategy 11: *Key words (in the field of "Title, Abstract or Keywords"): physician* + Electronic Medical

Record*

*Search Strategy 12: *Key words (in the field of "Title, Abstract or Keywords"): physician* + Electronic Health Record*

### Selection Criteria

Studies identified using the above strategies had to further meet the following selection criteria to be included in the literature review: 1) articles written in English, 2) article solely focused on EMR or EHR, not involving other electronic systems used in medical practices, 3) articles related to barriers linked to physicians (medical specialists, general practitioners), not to other medical staff, 4) based on empirical studies and published in scientific journals. As such, articles not specifically focusing on EMR/EHR (for example on IT systems or computerization) were excluded. Further, articles whose target groups were practices or clinicians were excluded because these articles not only covered physicians but also nurses, physician's assistants and other staff. The articles first identified in the reference lists of the papers found through the database searches were assessed using the same criteria.

### Data Analysis

For each of the studies that had survived this filtering, the research approach was first assessed. If it was a qualitative study, the number of cases and the methods used in data collection were identified. If it had used a quantitative approach, information concerning the sample size was sought. At the same time, the countries and the clinical areas of the included studies were recorded. Secondly, the empirical results of the studies related to expected or experienced barriers were summarized for further analysis. Further, the barrier focus of each study was deduced from the title or the abstract of the article.

## Results

### Included Studies

**Figure 1:**
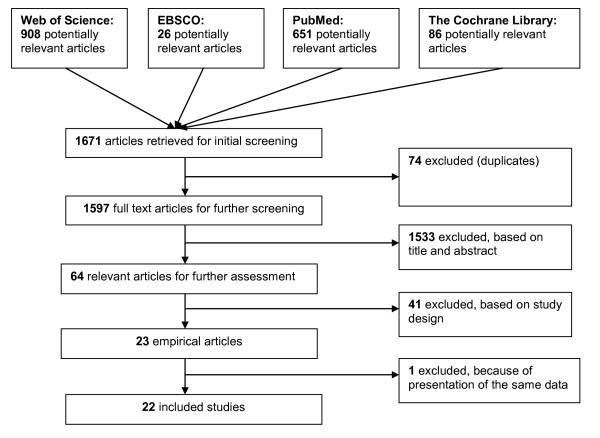
**Flow chart of study selection process**.

By applying the different database search strategies, 1671 articles were identified (including several duplicates of articles appearing in more than one database), see Figure 1.

After an initial screening carried out by two independent student researchers, 72 articles were duplicates. Of the remaining 1597 articles, 1533 were excluded because they did not meet, based on the content of their titles and abstracts, one of selection criteria 2), 3), and 4). Disagreements about exclusions between the two student researchers were resolved through discussions with one of the authors of this paper. Of the 64 articles remaining, 41 were commentaries or literature reviews, therefore lacking fresh empirical data and thus excluded. Of the 23 empirical studies left, one was presenting the same data with similar analyses and, as the relevance was minor, only one was retained. No additional relevant articles were found within the reference lists, as those meeting the selection criteria were already included. Thus, we ended up with 22 articles that met the inclusion criteria, and these were further analyzed by the two student researchers and both authors. Table 1 below lists the selected articles and the identified barriers.

**Table 1:** Overview of Included Studies

Author	Country/region of data Collection	Clinical Area	Expected or Experienced Barriers	Type of Research (Qualitative/Quantitative)	If qualitative		If quantitative		Focus
						
					Number of cases/number of physicians involved	Methods for Data Collection	Sample size/sampling strategy/response rate	Data Collection Method	
Jha et al. [10]	U.S.A Massachusetts	All specialties	1) computer skills of physicians and/or staff, 2) computer technical support 3)lack of time to acquire system knowledge 4)start-up financial costs 5)ongoing financial costs 6)training and productivity loss 7)physician skepticism 8)privacy or security concerns	Quantitative			1884 stratified/random sample/ 71%	Question-naire	All barriers

DesRoches et al. [6]	U.S.A	Direct patient care	1) capital costs 2) not finding a system that meets the needs 3)uncertainty about return on investment 4)concern over system obsolescence	Quantitative			2758/ all physicians who provide care from AMA/ 62%	Survey	All barriers

Menachemi et al. [11]	U.S.A Florida,	Ambulatory care	1) upfront cost of hardware/software 2)ongoing maintenance costs 3)inadequate return on investment 4)additional time for data entry 5)no time to acquire/implement such a system 6)slows the work of physicians 7)temporary loss of productivity and/or revenue 8)no time to learn how to use 9)disrupts workflow and/or office's physical layout 10)lack of uniform data standards within the industry 11)temporary loss of access to patient records if computer crashes or power fails 12)products do not meet needs 13)physicians and/or staff lack technical knowledge 14)privacy/confidentiality concerns	Quantitative			4203/ stratified random sample/ 28.2%	Question-naire	All barriers

Randeree [8]	U.S.A	Orthopedics	1) cost 2)increase in staff workload 2)supplier presence 3)vendor trust 4)customizability 5)reliability	Qualitative	3 cases 13 phys	Interview			All barriers

Miller et al. [9]	U.S.A	Primary care	1) high initial financial costs 2)slow and uncertain financial payback 3)high initial physician time costs 4)complexity of technology 5)more time to learn how to use 6)difficult complementary changes 7)inadequate support 8)inadequate electronic data exchange 9)lack of project champions 10)lack of incentives	Qualitative	90 phys	Interview			All barriers

Simon et al. [12]	U.S.A Massachusetts	Primary care	1) start-up financial costs 2)ongoing financial costs 3)loss of productivity 4)lack of computer skills 5)lack of technical support 6)lack of uniform standards 7)technical limitations of systems 8)concerns about privacy and security 9)organizational size 10)organizational type 11)lack of support from other organizations	Quantitative			1181/ stratified random sample/ 71%	Question- naire	All barriers

Davidson et al. [5]	U.S.A	All specialties	1) cost 2)reluctance to replace a recently acquired system in order to integrate with an EHR 3)uncertainty about the vendor 4)work to convert the records 5)waiting to see if subsidies are offered	Qualitative	26 phys	Interview			All barriers

Pizziferri et al. [21]	U.S.A	Outpatient primary care	1) more time per patient	Qualitative	5 cases 16 phys	Observation			Time

Shachak et al. [25]	Israel	Primary care	1) lack of proper typing ability 2)disturbing patient-doctor communication	Qualitative	25 phys	Interview + Observa-tion			Patient-doctor communication

Walter et al. [22]	U.S.A	All specialties	1) professional autonomy	Quantitative			203/ randomly selected/ 34%	Question- naire	Autonomy

Burt et al. [24]	U.S.A	All specialties	1) organizational factors of the practice	Quantitative			3360/ probability sample/ 56%	Question-naire	Organiza-tional factors

Simon et al. [27]	U.S.A Massachusetts,	All specialties	1) organizational size 2) patient privacy concerns 3)lack of time	Quantitative			1345/ random sampl/e 71.4%	Question-naire	All barriers

Earnest et al. [23]	U.S.A	Clinic for congestive heart failure	1) privacy concerns 2)disturbing patient-doctor communication	Qualitative + Quantitative	7 phys	Interview		Question-naire	All barriers

Loomis et al. [13]	U.S.A Indiana,	Family care	1) concerns about data entry 2)costs 3)security and confidentiality 4)lack of belief in EMRs	Quantitative			618/ all active members of IAFP/ 51.7%	Question-naire	All barriers

Laerum et al. [18]	Norway	All specialties	1) access to computers 2)computer literacy 3)not flexible 4)traditional work routines	Quantitative			227/ random, very small and very large excluded/ 72%	Question-naire	All barriers

Ludwick et al. [19]	Canada Alberta	Primary care	1) training and after-sales experience with the vendor 2)technical support from the vendor 3)extra time needed for data entry 4)time constraint in procurement and implementation 5)computer skills of the physicians 6)disruption of the flow of information	Qualitative	9 phys	Interview			Sociotechnical barriers

Valdes et al. [14]	U.S.A	Family care	1) cost 2)work slowed 3)business failure 4)security concerns 5)standardization	Quantitative			5517/ all members of AAFP with email addresses/ 15.5%	Question-naire	All barriers

Vishwanath et al. [15]	U.S.A	All specialties	1) cost issues 2)ROI issues 3)lack of hardware 4)lack of financial incentives 5)logistics and regulatory issues 6)concerns over customization 7)herd mentality/social influence 8)need for control 9)concerns over adopting new technology 10)lack of community level participation	Concept mapping (85 physicians)					All barriers

Meade et al. [16]	Ireland	All specialties	1) lack of time 2)cost 3)poor training 4)absence of computer skills 5)lack of financial resources 6)poor typing ability 7) fail to find a suitable system	Quantitative			2951/ all Irish GPs/ 64%	Question-naire	All barriers

Kemper et al. [17]	U.S.A	Pediatric	1) expense of implementation 2)inability to find an EHR that meets the requirements 3)inability to interface with existing systems 4)system downtime 5)lack of a clear return on investment 6)transience of vendors 7)increase in physicians' workloads 8)no improvement in patient care or clinical outcomes 9)increase in staff workload 10)staff have inadequate computer skills 11)interference with doctor-patient relationship 12)patient confidentiality	Quantitative			526/ random sample/ 58%	Question-naire	All barriers

Terry et al. [20]	Canada Ontario	Primary care	1) time constraints to learn EHR 2) absence of a champion or problem solver 3) too low level of computer experience	Qualitative	50 physicians Synthesis of three studies	Interview Focus group			All barriers

Reardon & Davidson [26]	USA Hawaii	Small practices, majority primary care	1) too little growth and expansion in order to invest in learning, uncertainty in ROI 2) too little knowledge and skills related to EHR 3) too much heterogeneity of organizational knowledge and activities related to EHR	Quantitative			567/practices focus on small practices/ 23%	Question-naire	Organizational learning barriers

Of these 22 studies, 13 were quantitative, 7 qualitative, 1 mixed qualitative-quantitative research, and 1 concept-mapping research. Only four of the studies were executed outside the USA, but many different clinical areas were included. As this breakdown indicates, the majority of the researchers had opted for quantitative research methods (questionnaires) to explore the barriers facing physicians in EMR adoption. This approach enables researchers to reach many physicians and cover a wide spectrum. This is beneficial in developing a general impression of how physicians view EMR adoption and the associated barriers.

### Taxonomy of Barriers to Electronic Medical Records

An overview of all the barriers mentioned in the 22 included studies was shown in Table 1. In order to have a general impression of the barriers to EMR adoption from the physician perspective, barriers linked to similar problems were grouped into a single category. In so doing, all the barriers listed in Table 1 were placed within one of eight categories (A - G), each of which included relevant sub-categories. Most of the studies had aimed to identify all relevant barriers and did not focus on a specific barrier in advance. These individual categories are discussed below.

#### Category A: Financial

The "Financial" category of barriers includes those related to the monetary issues involved in implementing EMRs. The monetary aspect was an important factor for many physicians. The questions commonly facing physicians are whether the costs of implementing and running an EMR system are affordable and whether they can gain a financial benefit from it. The costs of an EMR system can be divided into two: start-up costs and ongoing costs. Some researchers do not distinguish between specific kinds of costs in their studies, but it seems safe to assume that these two types of costs are included in these studies since implementing an EMR system is recognized as a complex process with several stages involving purchasing, coordinating, monitoring, upgrading, and governance costs. The financial barrier was the one most frequently mentioned in the 22 included studies. Based on the studies and the identified barriers, we broke this category down into four sub-categories as follows:

##### A1 High start-up costs

Start-up costs include all the expenditure needed to get an EMR system working in the physician's practice, such as the purchase of hardware and software, selecting and contracting costs and installation expenses. These costs seem to be in the range from $16,000 to $36,000 per physician, with EMR software costs alone typically $10,000 per physician [8,9]. Many researchers state that these costs are significant and therefore should be regarded as a high barrier to physicians adopting EMRs, especially for those without large IT budgets. Twelve of the 22 included studies emphasized that high start-up costs were a primary and major barrier to EMR adoption [5,6,8-17].

##### A2 High ongoing costs

In addition to the start-up costs, implementing an EMR system requires extensive commitment to system administration, control, maintenance, and support in order to keep it working effectively and efficiently. These costs include the long-term expenditures incurred in monitoring, modifying, upgrading and maintaining EMRs, which will be significant. Further, vendors charge a lot of money for after-sales service. All of these projected costs make physicians unwilling to adopt EMRs [5,6,8,10,11,13-16]

##### A3 Uncertainty over Return on Investment (ROI)

As implementing and maintaining EMRs is very costly, physicians worry that their practices will face substantial financial risks and that it could take years before they see a return on the investment [9]. According to Miller and Sim [9] "financial benefits vary greatly, from none in practices that made few work practice changes to more than $20,000 per physician per year in the few practices that eliminated most paper processes" [[9], p.119]. While EMR vendors claim that the benefits outweigh the costs, physicians remain to be convinced [6,8,9,11,14,15,17].

##### A4 Lack of financial resources

Only one paper directly pointed to a lack of financial resources/funds as a barrier to EMR adoption (Meade *et al*., 2009). However, the high start-up and ongoing costs of implementing an EMR system (Barrier A1 & A2) can result in problems finding sufficient financial resources in a medical practice. As these costs are very high, there can be inadequate financial resources to cover them, especially in small and medium practices with low IT budgets.

#### Category B: Technical

Electronic Medical Records are hi-tech systems and, as such, include complex hardware and software. A certain level of computer skills by both suppliers and users (the physicians) is required. Further, there are still some technical problems with EMRs, which lead to complaints from physicians, and they need to be improved. Therefore, barriers exist related to the technical issues of the systems, the technical capabilities of the physicians and of the suppliers which are grouped in this second category.

##### B1 Physicians and/or staff lack computer skills

Many researchers, based on their surveys, have concluded that physicians have insufficient technical knowledge and skills to deal with EMRs, and that this results in resistance [10-12,16-20] Meade *et al. *[16] observe in this context that most of the current generation of physicians in Ireland received their qualifications before IT programmes were introduced. EMR providers appear to underestimate the level of computer skills required from physicians, while the system is not only seen as but in practice actually is very complex to use by these physicians (Barrier B3). Further, good typing skills are needed to enter patient medical information, notes and prescriptions into the EMRs, and some physicians lack them. Shachak *et al.*[21] found that EMR use introduces a new type of medical errors: typos. Further, it is not only the physicians but also other staff at medical practices who lack adequate computer skills. This general lack of skills hinders the wide adoption of EMRs.

##### B2 Lack of technical training and support

Many physicians complain of poor service from the vendor, such as poor follow-up with technical issues and a general lck of training and support for problems associated with the EMRs [8]. Ludwick *et al. *[19] similarly note that physicians struggle to get appropriate technical training and support for the systems from the vendor. As physicians are not technical experts (Barrier B1) and the systems are inherently complicated (Barrier B3), physicians perceive a need for proper technical training and support, and are reluctant to use EMRs without it. Simon *et al. *[13] found that two-thirds of physicians indicated a lack of technical support as a barrier to them adopting EMRs, while Ludwick *et al. *[19] noted that some physicians reported a lack of access to vendor technical support.

##### B3 Complexity of the system

Miller and Sim argue that most physicians "consider EMRs to be challenging to use because of the multiplicity of screens, options and navigational aids" [[19], p.120] The complexity and usability problem associated with EMRs results in physicians having to allocate time and effort if they are to master them. Physicians have to learn how to use the EMR system effectively and efficiently (Barrier C2) which they may see as a burden. It is also possible that a lack of skills (Barrier B1) leads the physicians to regard the EMR system as extremely complicated. The complexity of the EMR system also leads to barriers in the "Time" category discussed below.

##### B4 Limitations of the system

Some physicians worry that EMRs are machine-based systems, made and programmed by IT companies. They are concerned that under certain circumstances, or as time passes, the systems will reach their limitations, become obsolete and will no longer be useful [6,12].

##### B5 Lack of customizability

According to Randeree, "customizability refers to the ability to be adapted of the technology system that fails to conform to specific needs of the user applications" [[8], p.494]. Mny surveys show that one reason why physicians do not adopt EMRs is that they cannot find a system that meets their special needs or that they can utilize to meet their requirements [6,8,9,13,15,17]. Some physicians may also use this lack of customizability as a way to avoid admitting to other reasons for avoiding EMRs (such as Barriers B1, C2, C3, and D2). However, it does seem that more effort is required from the vendors of EMRs to increase their customizability. However, such customer services will increase the costs to practices of implementing EMRs; potentially erecting financial barriers (Barriers A1, A2, and A4).

##### B6 Lack of Reliability

"Reliability is the dependability of the technology systems that comprise the EMRs" [[8], pp.493-494]. High reliability is very important for a system dealing with patient information, and many physicians are concerned about the temporary loss of access to patient records if computers crash, viruses attack or the power fails [8,11,17]. Moreover, some fear the possibility of record loss due to an unknown technical defect in the system. Further, reliability problems will lead to financial loss, such as in the form of an increase in ongoing costs (Barrier A2).

##### B7 Interconnectivity/Standardization

EMR hardware and software cannot be used straight "out of the box", it has to interconnect with other devices that "complement" the EMR system and help to generate benefits. Among physicians in medical practices that have implemented EMRs, such interconnectivity problems are a well-recognized obstacle to the wide adoption of EMRs. In essence, EMRs are not compatible with the existing practice systems, and physicians are reluctant to get rid of functional systems in order to have an integrated system including EMRs [5,17]. Further, based on a survey, Valdes *et al. *[14] concluded that there were more than 264 unique types of EHR/EMR software implementations in use. The format of data varies among the different software packages and systems, in large part due to the lack of consistent data standards within the industry, and this makes data exchange difficult if not impossible between systems [9,11,12,15-17]. This problem is more acute in smaller practices than in larger ones because of the relatively limited organizational resources such as expertise and experience.

##### B8 Lack of computers/hardware

The use of EMR systems requires a sufficient quantity of hardware in practices, including computers, phone lines and internet connections. Some researchers state that some practices lack these 'basic' facilities/hardware needed to support EMR implementation [15,18] and that this issue blocks the widespread adoption of EMRs. Further, in such practices, the start-up costs associated with setting up EMRs will be higher as more resources are needed. Both issues are often seen as barriers within the "Financial" category (Barriers A1 and A4) and, as a consequence, only a few researchers directly refer to the unavailability problem of computers/hardware.

#### Category C: Time

A fluent workflow is very important to the work of physicians. The introduction of EMRs will slow a physician's workflow, as it will always lead to additional time being required to select, implement and learn how to use EMRs, and then to enter data into the system. As a result, their productivity will be reduced and their workload will be increased. This can cause financial problems, such as a loss of revenue.

##### C1 Time required to select, purchase, and implement the system

It has been found that physicians opt not to invest time in system selection and procurement [10,11,16,19] as they think they should spend their time and effort on patients, rather than on selecting and contracting an EMR system, which is not regarded as part of their daily working practice. However, there is no clear statement that physicians should be responsible for this work. Therefore, whether physicians investing time in selecting, purchasing, and implementing is really a barrier depends on the quality of project management during the EMR implementation process.

##### C2 Time to learn the system

Alongside the barriers introduced in the "Technical" category (the lack of computer skills (Barrier B1) and the complexity of the EMR system (Barrier B3)), physicians also need to spend time and effort on learning how to use an EMR system. However, "the demands and pressures of delivering office-based care may not afford them the time to learn the system" [12]. Given this situation, they report that they lack the time to learn, as it would slow their workflow and increase their workload. However, other researchers argue that mastering an EMR system will help physicians to work more efficiently [16]. Maybe, further benefit-effort analyses are required to demonstrate the value of learning and mastering the system.

##### C3 Time required to enter data

It is perhaps surprising that many researchers conclude that data entry is a problem for physicians using EMRs [11,13,14,17-19]. In Loomis's (2002) research, more than half of the EMR users stated that data entry was both cumbersome and time-consuming. As such, data-entry is a widely experienced barrier among physicians. It can be related to the complexity of the system (Barrier B3), or the inability of physicians to properly handle the system (Barrier B1), both mentioned within the "Technical" category.

##### C4 More time per patient

Many physicians report that using EMRs will take more time for each patient than using paper as, in some situations, it might be more convenient and efficient to use paper records during the clinical encounter [18]. If using EMRs, physicians may have to stop halfway through a consultancy in order to enter information on patients or type a prescription, and this will disrupt the flow. Additionally, the fact that physicians are slow in typing and entering data (Barrier C3) will cost more time for each patient visit than before. Focusing on this issue, Pizziferri *et al. *[21] carried out a time and motion study on physicians' time utilization before and after implementing an EMR system and found that most physicians were able to avoid "sacrificing time with patients or overall clinic time, but they do spend more time on documentation outside of clinic sessions" [[21], p.183]. The same study also showed that using EMRs does increase a physician's workload although, as a note of caution, we would add that there are no other studies focusing on this issue to confirm these findings. Given the technical problems noted earlier, such as physicians' lack of computer skills (Barrier B1) and the complexity of EMR systems (Barrier B3), an EMR system's ease of use is a key element in the efficiency and acceptance of such systems.

##### C5 Time to convert patient records

Implementing an EMR means switching from paper-based to electronic-based systems, and this involves transferring records between the two systems. Some physicians regard the task of record conversion as their own responsibility as "they are only comfortable with the summaries they themselves would make of handwritten notes, histories, and so on" [[5], p.25]. They are aware of the time and cost burden associated with record conversion, which may outweigh any acknowledged potential benefits of EMRs. As a result, the time required to convert records is considered as a barrier to the integration of EMRs in medical practices.

#### Category D: Psychological

Physicians have concerns regarding the use of EMRs that are based on their personal issues, knowledge, and perceptions. Their perceptions of the questionable quality improvement associated with EMRs and worries about loss of professional autonomy come within this category.

##### D1 Lack of belief in EMRs

According to Kemper *et al. *[17] more than half (58.1%) of the physicians without an EMR doubt that EMRs can improve patient care or clinical outcomes. Other researchers have stated that those who are unwilling to use such a system are skeptical about claims that EMRs can successfully improve the quality of medical practices [10,12,15].This creates a personal resistance to the adoption of EMRs. However, this is very much a perceived barrier to EMRs, there is a lack of valid statistical data and success stories about EMRs available to non-users. Clearly, implementing EMRs does mean a change in working styles for physicians and, initially, people are generally afraid of change and doubt its necessity. Further, the physicians' skepticism and negative perceptions of EMRs are affected by the social influences around them, and this will be discussed under the "Social" barrier category.

##### D2 Need for control

Professional autonomy plays a very important role in the working practices of physicians. Professional autonomy is defined as "professionals having control over the conditions, processes, procedures, or content of their work" [[22], p.207], which will not be possessed or evaluated by others. With the implementation of EMRs, physicians are concerned about the loss of their control of patient information and working processes since these data will be shared with and assessed by others. Walter and Lopez [22] concluded that physicians' perceptions of the threat to their professional autonomy are very important in their reaction to EMR adoption. Very few studies have really considered this problem (two articles among the twenty included studies) and offered suggestions to overcome it [15,22].

#### Category E: Social

Rather than working alone, physicians in medical practices work together and cooperate with other parties in the healthcare industry, such as vendors, subsidizers, insurance companies, patients, administrative staff, and managers. The decision-making process over EMR implementation by physicians is influenced by these parties and will also affect the relationship between physicians and patients. The relationships between a physician having to make an EMR decision and these other parties can create what can be categorized as "Social" barriers.

##### E1 Uncertainty about the vendor

As observed in the "Technical" category, a lack of technical training and support from vendors has been reported as a barrier to the adoption of EMRs by physicians. Therefore, the quality of vendors of EMR systems is crucial for the acceptance of EMRs. EMR systems are still relatively new in the marketplace [8]. The lack of suitable vendors reflects an immature industry, without sufficient viable products or competitors able to offer better services, and without enough information on vendors to enable an informed decision. Physicians are concerned that vendors are not qualified to provide a proper service, or will go out of business and disappear from the market, leading to a lack of technical support (Barrier B2) and a large financial loss (Barrier A3) [5,8,17]. Given the high costs of implementing EMRs, physicians are unwilling to enter this market without confidence in reputable and trustworthy vendors.

##### E2 Lack of support from external parties

Some researchers state that the reason why physicians have not yet adopted EMRs is a lack of involvement and support from external parties [5,12,15,23,24]. Davidson *et al. *[5] comment that physicians in small practices are waiting until the costs of adopting EMRs are covered by subsidies. Further, Simon *et al. *[13] noted that although the national agenda in the USA encouraged EMR adoption, one-third to one-half of physicians commented that their decision-making was affected by local and regional organizations that were not active in the EMR debate. Furthermore, insurance companies, which work closely with medical practices, lack specific actions and policies to support the use of EMRs [15] and this influences decisions by physicians on EMR adoption. Despite these indications, this issue has not been researched in detail and further studies with detailed explanations and analysis are needed to reach a better understanding of the role of external parties in physicians adopting EMRs.

##### E3 Interference with doctor-patient relationship

Only a few researchers have considered the possibility of interaction problems between doctors and patients when using EMRs. In Shachak's research [21], where this issue was considered, 92% of physicians felt EMR use did disturb communication with their patients. Physicians have to turn to the computer to complete electronic forms during the encounter, and this can be time consuming especially when they suffer from limited computer skills (Barrier B1). In the research by Ludwick *et al. *[19], some physicians reported that they sometimes stop using EMRs because hunting for menus and buttons disrupts the clinical encounter. According to Shachak *et al. *[25], using EMRs increases the average screen gaze time of physicians from 25% to 55% of the consultancy session, inevitably resulting in less eye-contact and less conversation with the patient. Alternatively, the physicians have to allow more time per patient which comes up against a barrier in the "Time" category (Barrier C4). Further, as some EMRs are patient-accessible, they might even distort the clinical encounter with more interference and distractions from the patient [23]. Thus, the traditional doctor-patient relationship will be changed by EMRs. However, whether this is really a problem for physicians and patients requires further empirical research since this issue has so far been largely neglected by most researchers.

##### E4 Lack of support from other colleagues

Physicians work cooperatively with other healthcare professionals, such as nurses and administrative staff, in medical practices. The lack of technical skills, complaints about workload increases, negative perceptions and resistance to using EMRs, all barriers which have been mentioned in previous categories, also apply to these colleagues. This leads to a lack of support from these colleagues, which impedes physicians in further adopting the system [8,15]. Again this is not widely acknowledged, and only these two publications in our survey saw this lack of support as a potential barrier to physicians adopting EMRs.

##### E5 Lack of support from the management level

Whether the management level supports the use of EMRs, and believes in the benefits of EMRs, has been found to influence the rate of EMR adoption by physicians [9,15,26]. However, most researchers do not consider this issue, or take for granted that managers will be committed to EMR implementation. This issue will be further discussed in the "Change Process" category (Barrier H4).

#### Category F: Legal

Electronic Medical Records deal with medical information on patients, and this should be treated as private and confidential. Physicians believe that keeping such information safe is very important because otherwise it could create legal issues. However, there is a lack of clear security standards which can be followed by those who are involved in the use of EMRs.

##### F1 Privacy or security concerns

Many researchers agree that the use of computerized EMRs is an issue that may have a negative effect on patient privacy [10,11,13-15,17,23]. Physicians doubt whether EMRs are a secure store for patients' information and records, and fear that data in the system may be accessible to those who are not authorized to obtain it. The consequent inappropriate disclosure of patient information might lead to legal problems. Furthermore, there is, in some countries, a lack of clear security regulations that could help ensure patient privacy and confidentiality. According to Simon *et al. *[27], physicians are more concerned about this issue than the patients themselves. Even among the physicians who do use EMRs, most believe that there are more security and confidentiality risks involved with EMRs than with paper records [13]. This shows that concerns about the privacy and security of patient data are experienced as a barrier to EMR usage.

#### Category G: Organizational

Physicians work in medical practices and hospitals, and the organizational characteristics of individual practices will be a factor in the adoption of EMRs. Physicians in different sizes and types of practices may well have different attitudes toward EMRs.

##### G1 Organizational size

Surveys by Miller *et al. *[9], Simon *et al. *[12], and Burt *et al. *[24] show that physicians in larger medical practices have a higher EMR adoption rate than those in smaller practices. It was also found that "physicians in larger practices are more likely to use available functions in their EMRs than those with EMRs in smaller practices" [[13], p.511]. One reason is that the physicians in larger organizations have more extensive support and training systems (Barrier B2) in the use of EMRs. Conversely, large organizations require more time to select, purchase and learn a system, convert and enter data, and for individual patient consultations (Barriers C1, C2, C3, C4 and C5).

The interconnectivity problems under the "Technical" category (Barrier B7) can be more easily solved by larger groups of physicians because they have more and stronger organizational resources, such as management expertise, practical experience, financial resources and support staff, than smaller groups [9]. Further, Randeree [8] notes that small practices have greater problems associated with the costs of EMRs than do large practices: small practices do not have large IT budgets to support the implementation and running of the system. Reardon *et al. *[26] show that small practices that are growing and expanding, using a practice management system and a variety of non-clinical information technology in their offices, may more readily overcome learning barriers related to the adoption of EMRs than those who lack these characteristics. Although several researchers have recognized differences in EMR adoption between small and large practices, few have analyzed the reasons for this, and further study is needed to fill this gap.

##### G2 Organizational type

Simon *et al. *[13] state that whether a practice is affiliated to a hospital is an important determinant of EMR adoption. According to Burt and Sisk [24], physicians who are employed by or contracted to a medical practice are more likely to use EMRs than those who own their own practices. Stand-alone physicians are most likely to cite high start-up and ongoing costs, a lack of technical training, lack of uniform standards, lack of time, lack of belief in EMR effectiveness plus confidentiality concerns as the major barriers to EMR adoption. Of the 22 studies included in our review, only two relate "organizational type" to the adoption of EMRs [12,24] and, given its apparent influence, further qualitative studies would be beneficial.

#### Category H: Change Process

Implementing EMRs in medical practices amounts to a major change for physicians who tend to have their own unique working styles that they have developed over years. This can make them unwilling to make or adapt to changes in their work. Therefore the change process in itself is a challenge as well as a problem. Problems that occur during the change process, such as the lack of a proper organizational culture, lack of incentives, individual and local resistance, lack of community level participation, and lack of leadership, fall within this category.

#### H1 Lack of support from the organizational culture

This issue is largely overlooked by researchers and implementers, although it is a very important part of a change process and interrelated with organizational working procedures. Of our reviewed studies, only Randeree [8] briefly mentions that the change of culture required to accompany a switch from the use of paper to an EMR system does not occur, and that this leads to slow adoption of EMR systems. Laerum *et al. *comment vaguely that "technology alone is not sufficient to achieve a well functioning electronic information system" [[18], p.1347]. To work successfully in new ways needs a change in certain organizational aspects. An EMR-friendly culture will support organization-wide use of EMRs. How to create an appropriate organizational culture for the use of EMRs is an important topic that deserves further research in learning how to implement EMRs successfully.

#### H2 Lack of incentives

EMRs have the potential benefit of improving the quality of medical care. However, unless physicians see some personal benefit from using EMRs, they will not be motivated to switch and will instead stick to their traditional working procedures. Miller and Sim [9] and Vishwanath *et al. *[15] concluded that unless physicians have some personal incentives during the implementation of EMRs, the adoption of EMRs will not reach the expected level. Interestingly, the incentives considered in the cited studies were largely financial ones and, to us, this seems an area worthy of wider investigation.

#### H3 Lack of participation

Only one article out of the 22 reviewed mentioned the problem of a lack of participation [15]. Potential participants include not only physicians, but also nurses, administrative staff, IT staff, and other organizational members. The wide adoption of EMRs will only be achieved if all organizational members participate in the use of EMRs. This is largely a problem because of the existence of other barriers, such as a lack of leadership (Barrier H4), a lack of supportive organizational culture (Barrier H1) and a lack of support from other colleagues (Barrier E4). The fact that this problem is mainly caused by other factors may be why most researchers do not refer to it specifically.

#### H4 Lack of leadership

From the project management perspective, project leaders or project champions play a critical role in the success of a project. In an EMR implementation project, project leaders/champions are the people who lead, encourage and support the change at the management level [20]. Provided they strongly believe that EMRs will bring benefits and quality improvement, they will be willing to bear the risks and costs in order to generate the benefits [9]. One important function of project leaders/champions is to motivate other members of a practice to participate in the change process. Miller and Sim [9] argue that practices without EMR champions may struggle to improve quality or see financial benefits from EMRs. As such, more attention should be paid to the role and influence of project leaders/champions in order to increase the adoption rate of EMRs.

## Discussion

The review of identified articles shows the wide range of possible barriers to implementing EMRs, and provides insight into the relationships between barriers. Table 2 shows that certain categories (A-Financial, B-Technical, and C-Time) are more often identified as barriers to EMR adoption than others (D-Psychological, E-Social, F-Legal, G-Organizational, and H-Change process). We deduce that the most frequently identified barriers are 'primary' barriers, i.e. they are the first to arise when physicians are faced with EMRs. In other words, EMRs are most often experienced by physicians as threats in financial, technical, or time-consuming senses. However, the research also indicates that there are 'secondary' barriers (categories D-H) which are sometimes subconscious, beneath the surface, and so not immediately mentioned. It is, however, important to be aware of these secondary barriers since the presence of primary barriers (A-C) might represent other, less visible, obstacles related to psychological (D), social (E) or change process (H) issues.

**Table 2:** Taxonomy of Barriers

Category		Barriers		References (Article No.)	References per category	References per barrier
A	Financial	1	High start-up costs	1/2/3/4/5/6/7/14/17/18/19/20	33	12
				
		2	High ongoing costs	1/2/3/4/6/7/14/17/18/19/20		11
				
		3	Uncertainty about Return on Investment (ROI)	2/3/4/5/17/18/20/22		8
				
		4	Lack of financial resources	19/21		2

B	Technical	1	Lack of computer skills of the physicians and/or the staff	1/3/6/9/15/16/19/20/21/22	41	10
				
		2	Lack of technical training and support	1/4/5/6/16/17/18/19/20		9
				
		3	Complexity of the system	3/5		2
				
		4	Limitation of the system	2/6		2
				
		5	Lack of Customizability	2/3/4/18/20		5
				
		6	Lack of Reliability	3/4/20		3
				
		7	Interconnectivity/Standardization	3/5/6/7/17/18/19/20		8
				
		8	Lack of computers/hardware	15/18		2

C	Time	1	Time to select, purchase and implement the system	1/3/16/19/27	28	5
				
		2	Time to learn the system	1/3/5/12/19/20/21		7
				
		3	Time to enter data	3/14/15/16/17/20		6
				
		4	More time per patient	3/5/6/8/15/16/17/20		8
				
		5	Time to convert the records	5/7		2

D	Psychological	1	Lack of belief in EMRs	1/18/20	5	3
				
		2	Need for control	10/18		2

E	Social	1	Uncertainty about the vendor	4/7/20	13	3
				
		2	Lack of support from external parties	6/7/18		3
				
		3	Interference with doctor-patient relationship	9/13/20		3
				
		4	Lack of support from other colleagues	4/18		2
				
		5	Lack of support from the management level	5/18		2

F	Legal	1	Privacy or security concerns	1/3/6/12/13/14/17/18/20/27	10	10

G	Organizational	1	Organizational size	4/5/6/11/12/22	8	6
				
		2	Organizational type	6/11		2

H	Change Process	1	Lack of support from organizational culture	4/15	8	2
				
		2	Lack of incentives	5/18		2
				
		3	Lack of participation	18		1
				
		4	Lack of leadership	5/18/21		3

From the results reviewed, we also saw that barriers within different categories or subcategories seemed to be interrelated. For instance, high start-up costs and ongoing costs (Barriers A1 and A2) lead to a lack of financial resources (Barrier A4); and a lack of adequate computer skills (Barrier B1) is the reason why physicians need a long time to learn the system (Barrier C2). Also the finding that organizational size (Barrier G1) seems to be an important factor, that should to be taken into consideration when determining the implementation process, seems to be an interesting topic for further investigation. In particular, barriers in the primary categories (A-Financial, B-Technical, and C-Time) vary significantly between small and large medical practices, with small practices facing greater difficulties in overcoming the financial, technical, and time barriers. Therefore, the focus and effort required to overcome these barriers to EMR implementation may differ depending on practice size.

**Figure 2:**
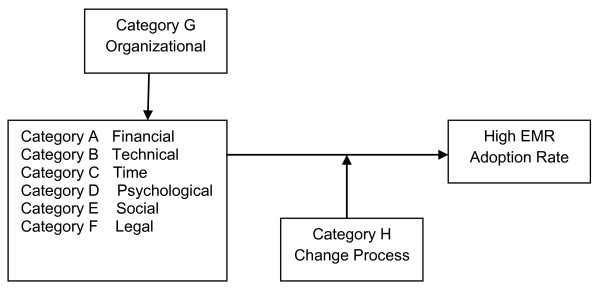
**Relationship among the barriers**.

Further, more attention should be paid to the last two categories of barrier: "Organizational" (Category G) and the "Change Process" (Category H). The barriers in these two categories are related to the characteristics of medical practices and the EMR implementation project itself. These barriers influence the other six categories of barrier at different times. First, the "Organizational" category barriers determine the relative importance of the other barriers even before implementation has started, as characteristics of a practice can affect the 'height' of certain barriers. For example, a small practice is expected to face greater difficulties in resolving financial issues than a large practice. Barriers in the "Change Process" category can mediate other identified barriers during the implementation process by restricting the ability to overcome them and achieve a successful EMR adoption. Ignorance of potential barriers in these two categories can lead to more serious barriers in Categories A-F. Therefore, Category G and Category H barriers can be seen as mediating factors in the success of an EMR project. The relationship among the barriers is illustrated in Figure 2.

Overcoming the barriers to physicians accepting EMRs is a complex process that needs support from several parties such as the government, insurance companies, vendors, managers, patients and especially the physicians themselves. This study suggests, however, that it is important for policy makers and implementers, such as hospital managers, project leaders and change managers, to understand which barriers are present in their specific situation in order to determine appropriate interventions that address those barriers. A topic for further research could be to develop a questionnaire or short-list to assess the "barrier-levels" in a particular practice. Based on the outcomes of such a scan, a customized implementation plan could be developed. Such a plan should regard the implementation of EMRs as a process of change and not just as overcoming barriers. Only then will the adoption of EMRs achieve the expected level. However, the current literature has only paid attention to the EMRs themselves, the financial and technical problems, and their influence on physicians' time and workload issues.

From this perspective, some barriers are within and others beyond the control of implementers. For instance, overcoming the high cost barriers, especially the purchase costs associated with EMRs, may require incentives from the government, such as low-interest loans or funding programs [28]. Anderson [28], when addressing privacy and security concerns (Barrier F1), similarly argued that national government action was required to develop and regulate a comprehensive set of national privacy laws on data protection. Many countries have already addressed these concerns through new laws and regulations. In the United States, for example, the Health Insurance Portability and Accountability Act (HIPAA) Privacy Rule offers federal protection for the privacy of personal health information. Other countries have comparable legal frameworks for dealing with medical data. The *'Study on the Legal Framework for Interoperable eHealth in Europe' *provides an overview of regulatory frameworks in European Union member states [29], but also notes that many privacy and security issues have to be resolved.

Interventions that are within the control of implementers may be directed at individual physicians, groups of physicians or all physicians who are intended to use EMRs. Our suggestion is that such interventions should be related to perceived barriers, and Table 3 proposes intervention strategies related to the eight categories of barrier.

**Table 3:** Perceived barriers and related possible interventions

Perceived barrier		Possible barrier-related intervention strategies
A	Finance	Provide documentation on return on investment. Show profitable examples from other EMR implementations. Provide financial compensation.

B	Technical	Educate physicians and support ongoing training. Adapt the system to existing practices. Implement EMR on a module-by-module basis. Link EMR with existing systems. Promote and communicate reliability and availability of the system. Acquire third party for support during implementation.

C	Time	Provide support during implementation phase to convert records and assist. Provide training sessions to familiarize users. Implement a user friendly help function and help desk. Redesign workflow to achieve a time gain

D	Psychological	Discuss usefulness of the EMR Include trial period. Demonstrate ease of use. Start with voluntary use. Let fellow physicians demonstrate the system. Adapt system to current medical practice.

E	Social	Discuss advantages and disadvantages for doctors and patients. Information and support from physicians who are already users. Ensure support, leadership, and communication from management.

F	Legal	Develop requirements on safety and security in cooperation with physicians and patients. Ensure EMR system meets these requirements before implementation. Communicate on safety and security of issues.

G	Organization	Redesign workflow to realize a better organizational fit. Adapt EMR to organization type. Adapt EMR to type of medical practice

H	Change process	Select a project champion, preferably an experienced physician. Let physicians (or representatives) participate during the implementation process. Communicate the advantages for physicians. Use incentives. Ensure support, leadership, and communication from management.

It can be observed that, in many countries, there are large-scale national initiatives to address some of the barriers. For example, in Canada an independent, federally-funded not-for-profit organization, Canada Health Infoway, has the task of accelerating the development of electronic health records across the country. Although this organization cannot enforce compliance, it has explicitly addressed technical and financial barriers. The development of a network of effective inter-operable electronic health record solutions across Canada is especially intended to remove technical barriers and to make the healthcare system more cost-effective.

In the US, the Hitech Act addresses financial barriers by offering incentive payments to those who adopt and use EHRs, and reducing Medicare payments to those who do not use them. Funding for EHR incentives has also been added to the Medicaid system. In order to receive EHR stimulus money, the HITECH act requires doctors to show "meaningful use" of an EHR system.

In Australia, Health*Connect *is a national strategy to establish and maintain standardized electronic health information products and services for healthcare providers and consumers. The strategy is a partnership between National, State, and Territory Governments which aims to leverage e-health systems in different parts of the health sector through a common set of standards such that health information can be securely exchanged between healthcare providers.

In many other countries, such as the United Kingdom, Denmark, the Netherlands, and France, comparable initiatives are being taken to develop a national electronic health infrastructure.

These initiatives are normally directed at developing a technical and legal structure for the exchange of medical information, and are often funded or sponsored by public resources. As such, many national EMR initiatives are directed mainly at overcoming barriers in financial, technical, and legal areas such as to create a context in which EMRs are available (technology), affordable (finance), and where their use is allowed (legal). However, the taxonomy of barriers developed in this paper indicates that physicians often experience other forms of barrier, and these need to be systematically addressed to realize high adoption rates. The interventions proposed in Table 3 are developed to facilitate policymakers and implementers at national, regional, and local levels to develop multifaceted, multilevel, and therefore more effective implementation strategies. Multifaceted because the strategies should address the various areas suggested by the taxonomy; and multilevel because these areas can potentially be addressed on national, regional, local, and individual levels, and at the same time. Reports on effective EMR implementations [30] support this argument and indicate that a broad range of situation specific interventions is a precondition for adoption. For example, McCarthy and Eastman [[30], pp. 14-15] state *'...it is important to stress that focusing on just one factor of implementation readiness is not sufficient*...*all factors work in concert to influence desired change associated with an EMR implementation. The factors work best as an integrated whole, overlapping and reinforcing each other*...'.

Notwithstanding the interesting results, this review and analysis has some limitations. Although we were very careful in developing our search strategy, given that using electronic patient information in healthcare processes is a broad field, we cannot guarantee that we did not miss any important findings. Second, although the taxonomy proposed in this study covers all the barriers previously identified, other taxonomies and categorizations could have been proposed to analyze and group the barriers. Third, we did not contact the authors of the studies to confirm that we had categorized their findings in appropriate ways. However, we do not think that contacting the authors would have changed the results of this study or the developed taxonomy. A final limitation of this study is that it is exclusively based on a literature review. The authors of the included studies will have had different purposes, and used different methods and interpretation means, in reaching their conclusions - conclusions which do not necessarily fully accord with those in this article.

## Conclusions

Despite the positive effects from using EMRs in medical practices, the adoption rate of such systems is still low and they meet resistance from physicians. In this article, based on a systematic literature review of 22 studies, barriers to physicians accepting EMRs have been identified. Further, these barriers have been sorted and grouped into eight categories. Of these, the "Organizational" and "Change Process" categories of barrier mediate the other six categories that contain "Financial", "Technical", "Time", "Psychological", "Social" and "Legal" barriers. In each category, several sub-categories were identified and analyzed.

The paper analyzes the reasons behind the relatively low adoption rate of EMRs among physicians. Implementing an EMR system clearly changes the workflow in a medical practice. Moreover, an EMR implementation is a major change that is felt throughout the practice; it demands complementary adjustments and innovation in other aspects such as to the structure and culture of a practice.

The findings of this study can be used as an overview of barriers that physicians might possibly see in the EMR implementation process and, as such, could be valuable for EMR policymakers and implementers. The study indicates that policymakers should be more aware of the reality that removing technical, financial, and legal barriers is not sufficient to ensure the realization of the promises of EMR. A range of other measures, as suggested in this study, may be needed if physicians are to come to a positive decision over using these systems in their daily practices. The study also suggests interventions that could be helpful to implementers in overcoming these barriers. However, it would be wrong to conclude that there is a "one way fits all" route. EMR implementers and change managers have to choose and decide on relevant interventions based on their actual conditions and situation. At the same time, they should consider the structures and conditions of the practices with which they are dealing - an interesting and challenging task.

## Competing interests

The authors declare that they have no competing interests.

## Authors' contributions

AB and MB jointly developed the concept underpinning the paper and the subsequent search strategy. They also interpreted and analyzed the data and developed the taxonomy. Both authors read and approved the final manuscript.

## Pre-publication history

The pre-publication history for this paper can be accessed here:

http://www.biomedcentral.com/1472-6963/10/231/prepub
